# Neurotrophic support by traumatized muscle-derived multipotent progenitor cells: Role of endothelial cells and Vascular Endothelial Growth Factor-A

**DOI:** 10.1186/s13287-017-0665-4

**Published:** 2017-10-13

**Authors:** Heidi R. H. Zupanc, Peter G. Alexander, Rocky S. Tuan

**Affiliations:** 1 Department of Bioengineering, Swanson School of Engineering, University of Pittsburgh, Pittsburgh, PA 15219 USA; 2 Center for Cellular and Molecular Engineering, Department of Orthopaedic Surgery, University of Pittsburgh School of Medicine, 450 Technology Drive, Room 221, Pittsburgh, PA 15219 USA

**Keywords:** Blast trauma, Muscle stem cells, Mesenchymal stem cells, Peripheral nerve repair, Neurotrophic factors, Paracrine mechanism, Endothelial cells, VEGF-A

## Abstract

**Background:**

Adult mesenchymal stem cells (MSCs) have been shown to increase nerve regeneration in animal models of nerve injury. Traumatized muscle-derived multipotent progenitor cells (MPCs) share important characteristics with MSCs and are isolated from severely damaged muscle tissue following surgical debridement. Previous investigations have shown that MPCs may be induced to increase production of several neurotrophic factors, suggesting the possible utility of autologous MPCs in peripheral nerve regeneration following injury. Recent findings have also shown that components of the vascular niche, including endothelial cells (ECs) and vascular endothelial growth factor (VEGF)-A, regulate neural progenitor cells and sensory neurons.

**Methods:**

In this study, we have investigated the neuroinductive activities of MPCs, particularly MPC-produced VEGF-A, in the context of an aligned, neuroconductive nerve guide conduit and the endothelial component of the vascular system. Embryonic dorsal root ganglia (DRG) seeded on poly-ϵ-caprolactone aligned nanofibrous scaffold (NF) constructs and on tissue culture plastic, were cocultured with induced MPCs or treated with their conditioned medium (MPC-CM).

**Results:**

Increased neurite extension was observed on both NF and tissue culture plastic in the presence of MPC-CM versus cell-free control CM. The addition of CM from ECs significantly increased the neurotrophic activity of induced MPC-CM, suggesting that MPC and EC neurotrophic activity may be synergistic. Distinctly higher VEGF-A production was seen in MPCs following neurotrophic induction versus culture under normal growth conditions. Selective removal of VEGF-A from MPC-CM reduced the observed DRG neurite extension length, indicating VEGF-A involvement in neurotrophic activity of the CM.

**Conclusions:**

Taken together, these findings suggest the potential of MPCs to encourage nerve growth via a VEGF-A-dependent action, and the use of MPC-CM or a combination of MPC and CM from ECs for peripheral nerve repair in conjunction with NFs in a nerve guide conduit. Due to the ease of use, application of bioactive agents derived from cultured cells to enhance neurotrophic support presents a promising line of research into peripheral nerve repair.

## Background

Within a critical time frame, nerve injury healing requires the careful orchestration of several independent but related processes [1]. When a nerve injury surpasses the length of a critical-sized defect, the ideal nerve healing rate of about one inch per month [2] may be limited by fibrotic scar formation. The target tissue slowly loses the ability to recruit the recovering nerve or encourage reinnervation [3]. While several strategies have been developed to stimulate bridging of the injury gap by accelerating nerve growth, the clinical treatment of choice, grafting of autologous nerve or blood vessels, promises no guarantee of recovery and requires precise and sometimes unattainable donor tissue geometry as well as a taxing, second surgery for tissue harvest [4]. When autologous donor tissue is unattainable, patients can be treated with synthetic, biodegradable nerve guides as a last resort, but such guides are currently associated with less favorable outcomes [5].

Exciting research in recent years has attempted to increase the neurotrophic potential of these nerve guide conduits, the most sophisticated of which attempts to mimic the physical, chemical, and temporal aspects of the native repair processes. The most basic function of these guides isolates the damaged nerve, concentrating neurotrophic signals from the distal stump while eliminating unwanted, scar-forming immune cell infiltration [6]. This cellular isolation must also allow constant nutrient exchange for the sensitive nerves. These competing concerns have been addressed in recent years by tightly controlling the porosity and thickness of nerve guide conduits [6]. Within that context, substrate patterning or the incorporation of aligned fibers mimic bands of Büngner formed by proliferating, reparative Schwann cells [7, 8]. Advancements in polymer chemistry and refinements of techniques in fibrous scaffold fabrication, such as electrospinning, allow the precise control of the fiber diameter, alignment, and degradation rate [9, 10] with the ultimate goal of complete scaffold replacement by native tissue. The clinically approved biomaterial, poly-ε-caprolactone (PCL) is a biodegradable synthetic polymer that can be easily utilized for the fabrication of electrospun micro- or nanofibers [11], with a degradation rate in vivo comparable to the rate of native tissue replacement [12]. Beyond providing a permissive architecture, PCL does little to increase nerve growth. To address this relative inertness, stem cells or growth factor augmentations of these guides represent a popular topic of research, including the incorporation of bioactive, neurotrophic factors normally secreted by Schwann cells [13]. These include nerve growth factor (NGF), brain-derived neurotrophic factor (BDNF), ciliary neurotrophic factor (CNTF), and glial cell-derived neurotrophic factor (GDNF). These factors can be tethered to or encapsulated within the nerve guide conduits for controlled release [8, 14, 15].

The bioactivity of exogenously delivered factors may degrade with time, and experiments have shown that direct transplantation of biologically responsive cellular growth factor sources, such as Schwann cells and Schwann cell-like bone marrow-derived mesenchymal stem cells (MSCs), can enhance nerve repair outcomes [16, 17]. It has been reported that nerve coculture with or treatment with conditioned medium (CM) derived from Schwann cells, Schwann-like MSCs, or undifferentiated MSCs positively influenced the length and density of neurons dissociated from chick embryonic dorsal root ganglia (DRG) [18, 19].

Ancillary to supporting the damaged nerve, MSCs are thought to signal the surrounding tissue and (possibly) invading immune cells with a resulting net positive effect on nerve defect healing [20, 21]. The use of allogeneic MSCs, while being tested for certain clinical therapies, faces intensifying scrutiny over their immunogenicity [22]. Current sourcing of sufficient numbers of autologous MSCs also involves invasive secondary procedures. A safe and efficacious autologous MSC isolation method, integrated with standard surgical treatment methods, is thus highly desirable.

Growing evidence suggests that skeletal muscle contains multipotent stem or progenitor cells [23]. MDSCs (muscle-derived stem cells), a preplating selected cell type related to pericytes or mesenchymal stem cells, have been shown to possess multilineage differentiation abilities [24]. Recent studies have shown that careful muscle tissue and cell isolation from the viable margins of surgical waste obtained during debridement of a blast-traumatized extremity yields a population of muscle-derived multipotent progenitor cells (MPCs) [25]. As described in this article, MPCs are defined by their isolation from traumatized muscle and rapid (24 h) adherence to tissue culture plastic. MPCs should not to be confused with MDSPCs, which are derived from nontraumatized muscle and adhere slowly to collagen-coated surfaces [26]. In addition to other functions, MPC neurotrophic activities and neurotrophic factor expression [27] in vivo are similar to those of widely known and clinically utilized bone marrow-derived MSCs [28], suggesting that MPCs might be an ideal candidate cell type for autologous nerve repair for victims who have suffered severe trauma and thus present readily accessible traumatized tissue specimens. To enhance the clinical utility of MPCs and the production of neurotrophic factors, MPCs can be neurotrophically induced following culture in a defined medium [27]; CM obtained following this induction can then stimulate neurite outgrowth in embryonic DRG [29]. Recent work also suggests that MPCs might convey neurotrophic benefits through indirect means, as MPCs exhibit the ability to control their local proteolytic microenvironment [30]. One manner of MSC tissue support might be through an association with vascular endothelial cells (ECs), both in the maintenance of a stem cell niche and in endogenous regenerative processes [31–33].

ECs are intimately connected with the development, growth, and continued health of nerves in the neurovascular unit [17, 34]. Neural and vascular cells often utilize similar molecular pathways for physical guidance [35], which could be exploited by MSC- or MPC-based therapies. There is ample evidence that the EC ligands, vascular endothelial growth factor (VEGF)-A and angiopoietin (ANGPT)-1, also exert positive effects on neurons [36]. VEGF-A receptor (VEGFR)2 is expressed by neurons [37], and recent clinical studies have reported that VEGF-A promoted anatomical and functional recovery of injured peripheral nerves in the avascular cornea [38], but the role of VEGF-A signaling with respect to MSC- and EC-associated peripheral nerve repair remains undefined.

Extrapolating from these findings, we hypothesize that MPCs positively modulate nerve growth activities and that this effect could be augmented through interactions with ECs and physical contact guidance. These interactions were investigated using the embryonic DRG model in two experimental set-ups: (1) treatment of DRGs cultured on tissue culture plastic with one or more cell-conditioned media (CMs); and (2) coculturing of DRGs and MPCs seeded onto an aligned nanofibrous biomaterial scaffold, simulating the microenvironment within a polymeric nerve conduit construct. The results reported here show that neurotrophic differentiation of MPC strongly affected DRG neurite extension on nanofibers, regardless of the presence or absence of ECs. Results from the CM investigations revealed that VEGF-A or VEGF-A-associated molecules in MPC-CM, derived from cultures under neurotrophic or growth conditions, were responsible for a large portion of the observed neurite length enhancement effect.

## Methods

### Nanofiber construct formation and assessment

Aligned nanofiber constructs (NFs) were deposited reproducibly on glass slides (Fisher, Pittsburgh, PA) by electrospinning, at ~ 15 kV difference from a 22-G needle placed 15 cm from a rotating mandrel, an 11.5% PCL (Sigma-Aldrich, St. Louis, MO) solution dissolved in 1:1 tetrahydrofuran:dimethylformamide (Sigma-Aldrich) [39]. NFs were prepared for cell culture by lining each edge with silicone sealant (RTV 734; Dow, Midland, MI); following an overnight cure, residual solvent was removed by storage for an additional night under vacuum with dessicant. Each slide was then disinfected with 70% ethanol (Fisher) and 10 min of UV-V light exposure (100–280 nm) (LEDwholesalers; Hayward, CA), Finally they were rinsed with Dulbecco’s phosphate-buffered saline (PBS; Invitrogen, Carlsbad, CA). After preparation, NFs were incubated in PBS with either serial applications of 100 ng/ml poly-l-lysine and 10 μg/ml laminin (Sigma-Aldrich) at 4 °C overnight, or with 10% fetal bovine serum (FBS; Invitrogen) at 4 °C 2× overnight. Fiber thickness and orientation were determined using scanning electron microscopy and plugins developed for ImageJ/Fij (BoneJ 1.4.0 [40]; Directionality 2.0 [41]).

### Culture of traumatized muscle-derived MPCs and ECs

Traumatized muscle-derived MPCs, isolated as described previously [28] (four male patients, average age 24 years), were expanded from low initial cell numbers to passage 5–8 in tissue culture-treated flasks (Fisher, Nunc, Rochester, NY) in growth medium (GM): α-minimum essential medium (MEM) + 10% FBS + penicillin-streptomycin-fungizone (PSF) + 1 ng/ml basic fibroblast growth factor (FGF)-2 (all reagents from Invitrogen). Human dermal microvascular ECs (HUVECs) were obtained from the Centers for Disease Control and Prevention (via Material Transfer Agreement) [33] and cultured on tissue culture-treated flasks in EGM-2MV medium (Lonza, Basel, Switzerland). At 90% confluency, cells were trypsinized (Invitrogen) and passaged at 1 × 10^6^ cells/T150 flask. All cells were cultured at 37 °C under 5% CO_2_.

Twenty-four hours after passage, CM was generated by culturing cells (denoted GM-MPC or EC) for 48 h in basal medium, containing Dulbecco’s modified Eagle’s medium (DMEM) + 1% insulin-transferrin-selenium-ethanolamine (ITS-X; Life Technologies, Carlsbad, CA) + penicillin-streptomycin (PS; Invitrogen). CM was denoted MPC-CM, EC-CM, or acellular control when derived from MPCs, ECs, or cell-free basal medium incubated with the same substrate, respectively. Following harvest, CM was prepared by centrifugation at 200 g, stored frozen at –80 °C until use, and thawed and centrifuged again just prior to use.

### Neurotrophic induction

MPCs were seeded at 1 × 10^3^ cells/cm^2^ on tissue culture plastic or NFs and differentiated neurotrophically using a modified 10-day protocol [27, 29, 42]. Twenty-four hours after seeding, MPCs were incubated with GM supplemented with 10 mM β-mercaptoethanol (Sigma) for 24 h, followed by 48 h in GM supplemented with 10 mM β-mercaptoethanol + 35 ng/ml retinoic acid (Sigma). For the 6 following days, MPCs were incubated with neurotrophic medium (DMEM/Ham’s F12 + PSF (Invitrogen) supplemented with 2% FBS, 2% B-27 (Invitrogen), 6 mg/ml retinoic acid, 1 ng/ml FGF-2 (Sigma), 10 ng/ml platelet-derived growth factor (PDGF; Sigma), 150 ng/ml heregulin (an isoform of neuregulin-1; Sigma), and 10 μM forskolin (Sigma)).

Following neurotrophic differentiation, MPCs (designated nMPCs) were washed with PBS and either lysed with TRiZol, or incubated with basal medium (see above) for 48 h to produce conditioned medium (nMPC-CM). For DRG coculture experiments, tissue culture plastic-cultured nMPCs were trypsinized and transferred to DRG-containing fibers at a concentration of 1 × 10^3^ cells/cm^2^.

### EC/MPC culture on nanofibrous constructs

MPCs and ECs were seeded on FBS-coated NFs at various densities (cells/cm^2^) in cell-specific growth medium; medium was exchanged every 48 h. Cell viability was assessed by quantifying the number of fluorescent live (green) and dead (red) cells visible within each microscopy image after Live/Dead staining (Invitrogen). Fluorescent cells were visualized with an Olympus inverted microscope equipped with a motorized stage controlled through MetaMorph. Whole cell numbers were determined automatically for each image by setting thresholds for fluorescence intensity, cell size, and cell shape using custom macros written for ImageJ/Fiji [43]. Cell density was determined by scaling the number of live cells observed in each image to 1 cm^2^.

### Nerve DRG culture and confirmation of MPC/EC neurotrophic activity

DRGs were obtained via microdissection from incubation day 9 chicken embryos, and placed on poly-l-lysine- and laminin-coated (Sigma) tissue culture plastic [44, 45]. After 24 h in nerve growth medium (basic Eagle’s medium, 10% horse serum, 1 nM GlutaMax; Invitrogen) supplemented with NGF + epidermal growth factor (EGF) + PDGF, DRGs were cultured for 4 additional days in a combination medium of 1:1 CM:DRG basal medium. CM-supplemented media were exchanged daily.

To assess the effect of MPC or EC coculture on DRG neurite extension, freshly isolated DRGs were seeded on sterile, aligned poly-l-lysine- and laminin-coated NFs. After 24 h in nerve growth medium, trypsinized MPCs, nMPCs, ECs, or a 1:1 xMPC-EC mixture were seeded onto DRG-containing NFs at a final density of 1 × 10^4^ cells/cm^2^ (1 × 10^5^ per NF) in 1 ml of DRG basal medium. Control cultures were incubated in only 1 ml of DRG basal medium. All media were exchanged daily for 4 additional days, and DRG neurite outgrowth was assessed as described previously.

### Selective VEGF-A depletion

VEGF-A was selectively removed from thawed, centrifuged, and filtered CM using neutralizing antibodies (mouse IgG, mouse aVEGF-A; R&D, Minneapolis, MN) bound to SpinTrap Protein G beads (Sigma). Beads and bound antibodies/factors were removed from CM by centrifugation at 200 g for 5 min. Bound VEGF-A was eluted from the beads, and selective binding was confirmed via VEGF-A enzyme-linked immunosorbent assay (ELISA; R&D). VEGF-A-depleted or immunoglobulin (Ig)G-control CM were indicated with “+αVEGF-A” or “+IgG,” respectively.

### Confirmation of neurotrophic induction

Neurotrophic factor production/secretion into CM was assessed via sandwich ELISAs (R&D). RNA was isolated with TRiZol, reverse transcribed, and analyzed by reverse-transcription polymerase chain reaction (RT-PCR) for neural gene expression (Superscript III (Invitrogen) 18S rRNA, BDNF, CNTF, GDNF, Nestin, NGF, VEGF-A (Qiagen, Hilden, Germany)).

### Neurite outgrowth measurement

To assess the effect of CM or MPC/EC coculture on neurite outgrowth, DRGs were fixed and stained immunohistochemically for heavy neurofilament (NEFH; Sigma) (Secondary Alexa-Fluor antibodies; Invitrogen). DRGs were visualized with an Olympus inverted microscope equipped with a motorized stage controlled through MetaMorph. Resultant mosaic images were stitched using Grid/Collection Stitching (Fiji) [46]. Each image represented one replicate. The 10 longest NEFH-positive neurite extensions were measured from the geometric center of the original DRG cluster (ImageJ).

### Statistical analysis

All data are presented as mean ± standard deviation, unless noted. Statistical differences and *p* values were determined by one- or two-way analysis of variance (ANOVA) and Sidak’s or Tukey’s test, as appropriate.

## Results

### Neurotrophic support by MPCs and ECs cultured on tissue culture plastic

**Fig. 1:**
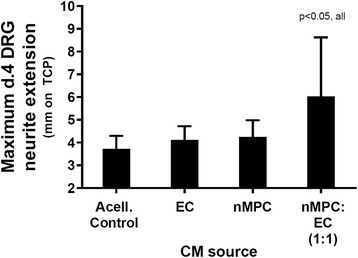
Neurite extension of dorsal root ganglia (DRGs) seeded on tissue culture plastic and cultured in the presence of conditioned medium (CM) from multipotent progenitor cells (MPCs) and/or endothelial cells (ECs). An increase in DRG neurite extension was detected as a synergistic effect of the mixed (1:1) CM derived from neurotrophically-induced MPCs (nMPC) and EC over the basal DRG media acellular control (Acell. control). *n* = 6; Tukey’s. Indicated *p* value applies to that condition versus all other conditions

To assess the relative trophic properties of the different cell types, conditioned medium (CM) from the various cell types or basal (control) medium was incubated with tissue culture plastic-seeded DRGs. In the presence of CM derived from ECs or neurotrophically induced MPCs, DRG neurite extensions increased slightly (but not significantly) above control lengths (Fig. 1). By contrast, DRG neurite extension lengths increased to almost twice that of the basal medium control in the presence of a combination of the CM from both cell types. This finding suggested that a combination of MPC and EC neurotrophic activities might better support neurite extension on a nerve guide conduit than either cell type in isolation.

### Nanofiber conduit (NF) fabrication

Efficient nanofiber-based physical guidance of neurite outgrowth requires the presence of appropriately sized (nanoscale) parallel fibers. Because batch-to-batch consistency of electrospun fibers is notoriously low [47], randomly selected scaffolds from multiple batches of NFs were examined using scanning electron microscopy. Fiber diameter was quantified through image analysis and suggested fairly consistent nanofiber diameter (580 ± 280 nm) and relatively good alignment (22 ± 17^o^ dispersion).

### Cell viability of effector cells on NFs

**Fig. 2:**
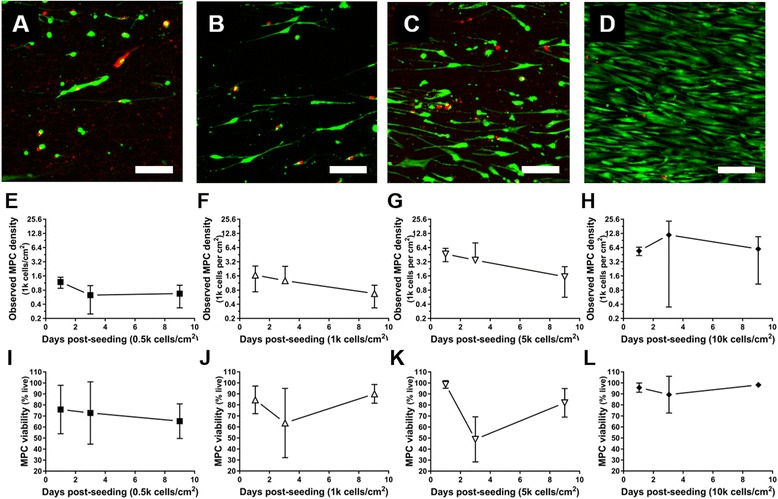
Viability and density of multipotent progenitor cells (*MPCs*) seeded on aligned NF scaffold. Edge-to-edge mosaic images of NF-seeded MPCs stained with Live/Dead (green/red) assay were obtained at 24 h post-seeding at (**a**,**e**,**i**) 0.5, (**b**,**f**,**j**) 1, (**c**,**g**,**k**) 5, and (**d**,**h**,**l**) 10 × 10^3^ (k) cells/cm^2^; *scale bar* = 100 μm. Evidence of NF-mediated cell alignment could be seen in the common orientation of the MPCs. **e**–**h** MPC density (presented as an exponential scale) as a function of culture time. MPC density scaled with initial seeding density at early time points (days 1 and 3), but eventually converged towards 1 k cells/cm^2^ by day 9, except for the 10 k cultures. Cell aggregation on the scaffolds resulted in extreme local variations in cell density. MPC viability (**i**–**l**) varied widely at early time points. By day 9, 65% or more cells remained viable, and viability increased with initial seeding density

To ensure that DRG-effector cell cocultures included sufficient space and nutrients for all cell types, including oxidative stress-sensitive nerve cultures [48, 49], effector cells were seeded on NFs and assessed for their long-term (>24 h) viability and density (cells/cm^2^). MPCs or ECs were initially seeded on 10 cm^2^ serum-coated NFs at varying densities (0.5, 1, 5, 10 × 10^3^ cells/cm^2^). Live cell density and percent viability were assessed daily using metabolic stain (Live-Dead stain) for the first 3 days and after an additional week in culture (Fig. 2), corresponding to the schedule of neurotrophic induction. Cells were cultured in their respective growth media to allow for maximum proliferation.

MPCs tolerated NF culture well, reaching an equilibrium density of approximately 1–5 × 10^3^ cells/cm^2^, but EC coverage and viability dropped steadily over the culture period. Cell aggregation contributed to large variations in within- and between-sample viability and density measurements.

Because of these findings, subsequent experiments involving NF scaffolds were examined after short culture (< 5 day) and at low-moderate initial cell densities (1 × 10^3^/cm^2^). To examine longer term (> 5 days) effects on neurite extension and neurotrophic differentiation, ECs and MPCs were cultured on tissue culture plastic.

### Influence of effector cell coculture on DRG neurite extension

**Fig. 3:**
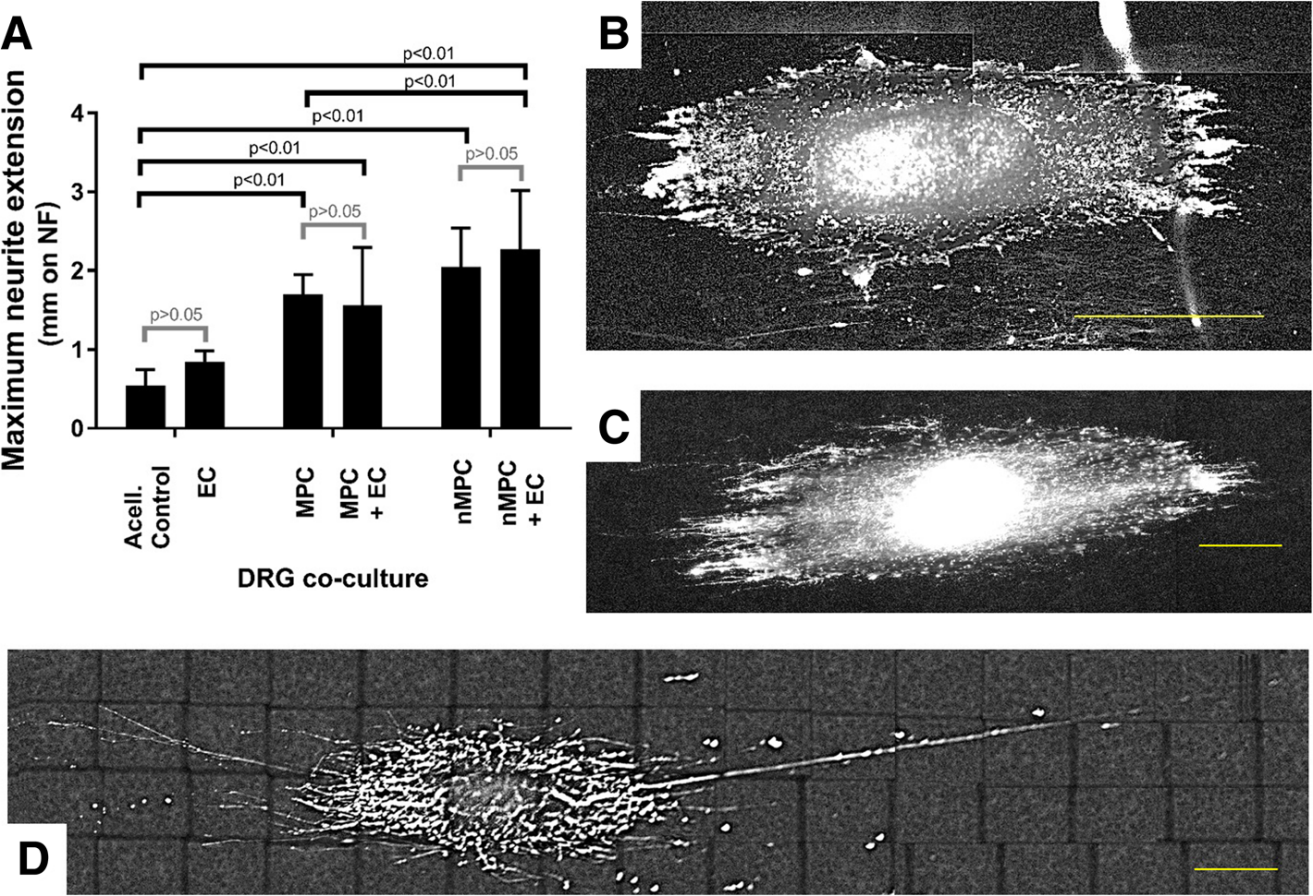
Neurite extensions of dorsal root ganglia (*DRGs*) seeded on nanofiber NF scaffold upon coculture with multipotent progenitor cells (*MPCs*) or combination of MPCs/endothelial cells (*ECs*) (1:1). The presence of MPCs, particularly after their neurotrophic induction, increased the length of DRG neurite extensions (**a**) over DRG control (acellular (*Acell.*) control/basal medium) cultures (**b**). EC coculture (**c**) slightly but not significantly increased DRG neurite extension length. MPC and neurotrophically-induced MPC (*nMPC*) coculture significantly increased neurite extension length when compared to the control. For xMPC-DRG coculture, EC coculture slightly but insignificantly increased neurite extension length. DRGs cocultured with both nMPC and EC (nMPC-EC-DRG cocultures; **d**) exhibited significantly longer neurite extensions than MPC-DRG or MPC-EC-DRG cocultures. **a** *n* = 4; Sidak’s *p* values as indicated. **b**–**d** *Scale bars* = 400 μm for representative NEFH-stained images

To assess the potential neurotrophic effect of the combination of MPCs/nMPCs and ECs (effector cells) in the context of a nerve guide, DRGs and effector cells were seeded onto NFs and cocultured. Prepared NFs were first seeded with DRGs. Twenty-four hours after DRG seeding on the NFs, cocultures were constructed by adding effector cells. Effector cells consisted of ECs, noninduced MPCs, nMPCs, and 1:1 EC-xMPC mix (co-effectors). Effector cells were added to the NFs at a final effector cell density of 1 × 10^3^ cells/cm^2^. NEFH-positive DRG neurite extensions were assessed after 4 days of coculture with daily medium changes (Fig. 3).

In the presence of MPC effector cells and regardless of the MPC neurotrophic induction, DRG neurite extensions increased. Specifically, DRG neurite lengths in nMPC-DRG cocultures were ~ 3- to 4-fold longer than DRG neurite lengths observed in control cultures (NF-seeded DRGs with no effector cells). This finding confirmed the functional utility of the neurotrophic differentiation protocol. The additional presence of ECs on the NFs caused a trend towards an increase in DRG neurite length with the longest extensions observed under co-effector coculture conditions. This effect suggests that a nerve guide conduit could promote nerve growth by incorporating a combination of MPCs and ECs.

### NF effects on effector cell neurotrophic activities

**Fig. 4:**
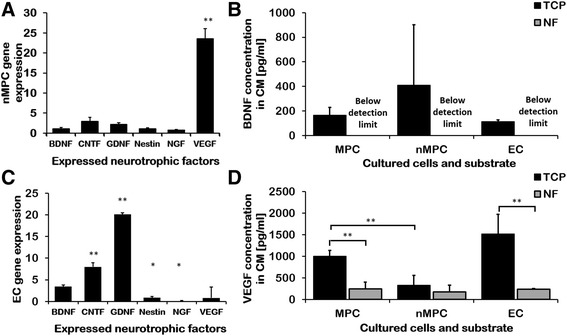
Expression and production of neurotrophically induced MPCs (*nMPC*) and endothelial cells (*EC*) cultured on tissue culture plastic (*TCP*) and nanofiber constructs (*NF*). **a**,**c** NF-cultured growth factor and neurotrophic marker gene expression, assayed by RT-PCR and normalized to TCP cultures: secreted brain-derived neurotrophic factor (*BDNF*) (**b**) or vascular endothelial growth factor (*VEGF*) (**d**) levels from cells seeded on TCP or NF as measured by ELISA. nMPC (**a**) and EC (**c**) neurotrophic gene expression was mildly or significantly increased by NF culture in almost all cases. CM from cultures on NF contained lower levels of secreted growth factors than conditioned medium (*CM*) from TCP-cultured cells (**b**,**d**). Noninduced multipotent progenitor cells (*MPCs*) secreted more VEGF than nMPCs (**b**,**d**). *n* = 6; Student’s *t* test *p < 0.05, **p < 0.01, versus controls (**a**,**c**) or indicated groups (**b**,**d**). *CNTF* ciliary neurotrophic factor, *GNDF* glial cell-derived neurotrophic factor, *NGF* nerve growth factor

To assess whether NF seeding might have altered MPC neurotrophic functions, MPCs were differentiated on NF and tissue culture plastic by seeding cells in induction medium onto each surface at densities of 1 × 10^3^ cells/cm^2^. Except for an increase in VEGF-A expression, neurotrophic gene expression of NF-seeded nMPCs did not change significantly compared to tissue culture plastic-seeded nMPCs (Fig. 4a and b).

However, pooled CM from NF-seeded nMPCs exhibited much lower factor concentrations than CM from similarly induced tissue culture plastic-seeded nMPCs. CM derived from tissue culture plastic-cultured nMPCs contained FGF-2 (130 ± 220 pg/ml, *n* = 6) and GDNF (30 ± 50 pg/ml) inconsistently, with multiple samples yielding undetectable amounts of each factor. Similar to BDNF, FGF-2 and GDNF could not be detected in CM derived from NF-cultured nMPCs, and secreted CNTF and NGF could not be detected in any samples.

When compared to culture on tissue culture plastic, EC culture on NF resulted in significant changes in EC neurotrophic gene expression (Fig. 4c and d). Despite these gene expression changes, only BDNF and VEGF-A could be detected in EC-CM from either substrate (tissue culture plastic or NF). NF culture severely reduced the level of neurotrophic growth factors measured in EC-CM. This observation and the above note of diminished cytokine and growth factor concentrations in NF-seeded cultures suggests that protein-scaffold interactions may restrict cytokine and growth factor release [50]. This likely explains the diminished relative neurite extension observed upon MPC or EC coculture with DRGs (Fig. 3) versus tissue culture plastic-derived CM neurotrophic support (Fig. 1).

### Mechanism of action of ECs and MPCs

**Fig. 5:**
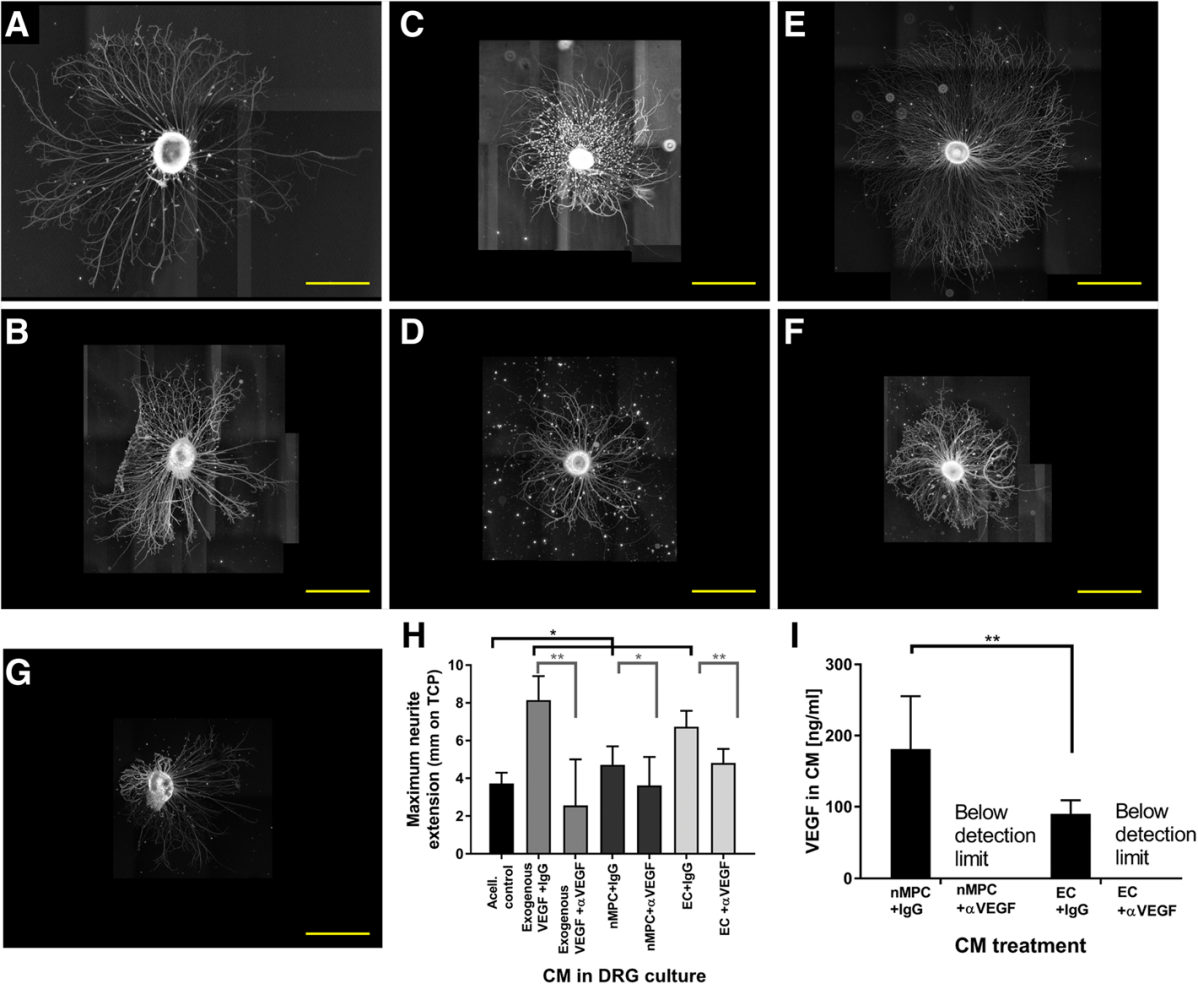
Vascular endothelial growth factor (*VEGF*)-A production by neurotrophically induced MPCs (*nMPC*) and endothelial cells (*EC*) positively affects neurite extension. **a**,**c**,**e** Dorsal root ganglia (*DRG*) neurite extensions in the presence of conditioned medium (*CM*) and immunoglobulin G (*IgG*) control antibody. **b**,**d**,**f** DRG neurite extensions in the presence of CM and α-VEGF-A antibodies (VEGF-A immunodepletion). Compared to nonspecific IgG control (**a**), selective removal of VEGF-A from a positive control consisting of exogenous VEGF-A (**b**) resulted in the loss of neurite growth induction, validating the effectiveness of VEGF-A immunodepletion. VEGF-A immunodepletion of CM derived from nMPC (**d**) and EC (**f**) decreased observed neurite extensions compared to IgG-treated mixtures (**c** and **e**, respectively), indicating that VEGF-A is an active component that contributes to both MPC and EC neurotrophic activities. **h** Quantification of neurite growth following depletion of VEGF-A. VEGF-A immunodepletion was associated with a significant decrease in CM-induced neurite outgrowth (one-way ANOVA, Tukey’s). **i** VEGF-A levels in CM measured by ELISA. VEGF-A was present in CM derived from both MPCs and ECs and could be removed by immunoprecipitation using VEGF-A-specific antibodies (versus control IgG antibodies). **a**–**g** Representative NEFH-stained images; *scale bar* = 1 mm. **h**,**i** *n* = 3; one-way ANOVA Tukey’s *p < 0.05, **p < 0.01

Interestingly, our analysis showed that VEGF-A, most commonly known for its role in angiogenesis and known to be expressed robustly by MPCs, was secreted by MPCs in copious amounts upon neurotrophic induction (Fig. 4b and d). The magnitude of secreted VEGF-A relative to other neurotrophic factors as well as the observation of VEGFR [51] on the surface of nerves suggested that it might play a more significant role in MPC-mediated neurotrophic activity than previously realized. To test whether VEGF-A was acting as a mediator of the MPC effect on DRG, VEGF-A was selectively depleted from nMPC-CM, EC-CM, and VEGF-A-spiked medium using anti-VEGF-A antibodies bound to Protein G beads. Effective VEGF-A immunodepletion was confirmed by ELISA (Fig. 5i). VEGF-A concentrations in control and IgG-treated medium were identical (~ 170 pg/ml), and VEGF-A concentrations in VEGF-A-depleted samples were below the detection limit. VEGF-A removal from CM derived from nMPCs (Fig. 5d) and ECs (Fig. 5f) significantly decreased the maximum observed length of DRG neurite extensions when cultured on tissue culture plastic (Fig. 5a–g), suggesting a dependence of the neurotrophic activity of both cell types on VEGF-A in addition to other MPC-secreted neurotrophic compounds.

## Discussion

We have previously reported that neurotrophic induction of MPCs enhances their production of neurotrophic factors, including BDNF and CNTF. Extending that work, results from this study show that nMPCs could support increased DRG neurite extension on nanofiber conduits with approved nerve guide materials. This support was partially mediated through soluble factors, including those factors found in conditioned medium and secreted during MPC-DRG coculture. This support was observed regardless of the substrate on which the DRGs were seeded (tissue culture plastic or nanofiber scaffolds (NFs)), despite adverse effects of the NFs on MPC viability and measurable growth factors in the CM. Due to high between-patient and between-experiment variability, consistent and significant differences in VEGF-A or BDNF concentrations in the CM were not observed. When compared to MPCs, nMPCs were associated with distinctly higher VEGF-A concentrations as well as consistent BDNF production, similar to results reported in other work [16].

Our findings show that the combination of nMPCs and ECs preferentially encourages neurite elongation (Fig. 1). Signaling molecules from each cell type work almost synergistically despite similar levels of known neurotrophic factors, BDNF and VEGF-A, in the respective CMs (Fig. 4b and d). This enhanced neurotrophic activity could be explained in part by a mixed population of cells in the isolated MPCs. A CD29^+^/CD34^+^ vascular endothelial progenitor cell (EPC)-like population has been noted within muscle isolates [52]; muscle-derived stem cells have additionally been observed to spontaneously express both vascular and neurotrophic markers upon culture with neurons [53], suggesting plasticity, cell fusion, or a culture of mixed cell types [53, 54].

For this investigation, nMPCs were investigated as local sources of neurotrophic factors in the context of an implanted NF. In keeping with previous studies, nMPC-CM was utilized without concentration [29]. Other researchers have reported greatly enhanced MSC trophic activities upon culture with cellulose fiber-concentrated CM [55], and it is possible that concentrating CM in future studies may yield stronger neural support.

To promote better DRG attachment and growth on NF substrate, laminin and lysine coatings were utilized in line with a growing consensus that many aligned fibrous structures bearing extracellular matrix (ECM) molecules are beneficial to the ultimate function of a nerve guide conduit [54]. Several groups have also investigated physical modifications to the PCL surfaces or chemical/ligand conjugation [12, 56] to better support initial nerve attachment and proliferation, and work is ongoing with coatings derived from various ECM molecules to increase MPC scaffold-based neurotrophic activity. Despite PCL NF culture of MPCs being unfavorable with respect to neurotrophic induction (Fig. 2), construct-seeded MPCs both survived on the NFs (Fig. 2) and encouraged increases in DRG neurite extension. Furthermore, the combination coculture of ECs and nMPCs encouraged the longest neurite extensions (Fig. 3). In recent work with adipose-derived MSCs implanted in rats, Kingham et al. showed that the neurotrophic differentiation of those cells simultaneously promoted both EC vascular and DRG nerve function [57]. Taken together, these findings suggest that by optimizing the interactions of MPCs and ECs preferential neural tissue growth could be achieved. Proteomic analysis of the secretomes of both cell types might elucidate synergistic neurotrophic interactions or potential antagonistic interactions between inhibitory factors in the CM of either the MPCs or ECs, the details of which warrant further study.

Neurotrophic and angiogenic factors have been found at widely varying levels within MSC- and muscle-derived stem cell secretomes, depending on the donor and tissue source [58, 59]. We therefore examined the potential involvement of VEGF-A in the neurotrophic potential of CM from ECs and nMPCs. Following immunodepletion of VEGF-A from CM, neurite extension length decreased when compared to the use of control antibodies, suggesting that VEGF-A is required for EC and nMPC promotion of neurite outgrowth (Fig. 5). This finding is consistent with other reports on the positive effect of VEGF-A on DRG neurite extension, and this work reports the first analysis of potential mechanisms for MPC enhancement of neurite extension.

## Conclusion

Taken together, these findings demonstrate that the neurotrophic activities of MPCs are enhanced by biological induction in vitro, CM combination with ECs, or coculture with adult ECs. VEGF-A played a role in the observed positive effect of MPC-CM on DRG neurite extension. Given its dual role as an angiogenic and neurotrophic factor, coupled with the observation of enhanced neurite extension under EC influence, VEGF-A should be incorporated in future NF-based cultures in an attempt to support both cell types. The unfavorable effect of the fibrous scaffold on MPC gene expression and measured protein secretion indicates that PCL, without appropriate ECM coating, is likely a suboptimal substrate for nMPCs. Testing of more cell-friendly substrates as well as coating the existing PCL scaffold structure with various ECM molecules are both ongoing and should yield useful information for the development of a regenerative construct for nerve repair.

## Abbreviations

BDNF: Brain-derived neurotrophic factor
CM: Conditioned medium
CNTF: Ciliary neurotrophic factor
DMEM: Dulbecco’s modified Eagle’s medium
DRG: Dorsal root ganglion
EC: Endothelial cell
ECM: Extracellular matrix
EGF: Epidermal growth factor
ELISA: Enzyme-linked immunosorbent assay
FBS: Fetal bovine serum
FGF: Fibroblast growth factor
GDNF: Glial cell-derived neurotrophic factor
GM: Growth medium
Ig: Immunoglobulin
MPC: Multipotent progenitor cell
MSC: Mesenchymal stem cell
NEFH: Heavy neurofilament
NF: Nanofiber scaffold
NGF: Nerve growth factor
nMPC: Neurotrophically induced multipotent progenitor cell
PBS: Phosphate-buffered saline
PCL: Poly-ε-caprolactone
PDGF: Platelet-derived growth factor
PSF: Penicillin-streptomycin-fungizone
VEGF: Vascular endothelial growth factor
VEGFR: Vascular endothelial growth factor receptor
xMPC: MPC or nMPC
